# Systemic Lupus Erythematosus Presenting with Massive Ascites: A Case of Pseudo-Pseudo Meigs Syndrome

**DOI:** 10.1155/2016/8701763

**Published:** 2016-06-05

**Authors:** S. McVorran, J. Song, V. Pochineni, A. Abrudescu-Opran

**Affiliations:** Department of Internal Medicine, Queens Hospital Centre, Queens, NY 11432, USA

## Abstract

The case presented is consistent with the phenomenon known as Pseudo-Pseudo Meigs Syndrome (PPMS). In it, we describe a young woman with newly diagnosed Systemic Lupus Erythematosus presenting with ascites, pleural effusions, and an elevated CA-125 level. Although rare, and of uncertain etiology, PPMS is becoming increasingly recognized in the literature. It should be considered as a differential diagnosis in such patients, along with the search for malignancy.

## 1. Introduction

Peritoneal serositis with ascites is an extremely rare manifestation of Systemic Lupus Erythematosus (SLE) [1]. It is usually described in established lupus patients and presents with mild to moderate, gradual onset, painless ascites. Massive (Grade III/IV) ascites has been rarely reported in SLE [2, 3] and when it does occur it typically accompanies active disease or results from nephrotic syndrome, protein-losing enteropathy, constrictive pericarditis, and conditions unrelated to lupus [1]. Peritonitis as the initial manifestation of SLE, without the above-mentioned factors, is exceedingly rare.

We describe here, therefore, a case of newly diagnosed Systemic Lupus Erythematosus in a young woman presenting with massive ascites. It is our hope that, in doing so, we can shed some light on this unique presentation.

## 2. Case Report

A 40-year-old Black female with no significant past medical history presented to Rheumatology Clinic, two weeks after an elective total abdominal hysterectomy and bilateral salpingectomy, with complaints of arthralgia for the past 6 months involving the proximal interphalangeal joints, wrists, knees, and ankles. She described morning stiffness of the hands, lasting for more than an hour, as well as swelling of the joints and Raynaud's phenomenon. In addition, she reported constitutional symptoms, such as fatigue, subjective fever, and chills. A history of photosensitivity was also present.

Since her surgery, she had an increase in abdominal girth with abdominal discomfort and exertional shortness of breath. Physical examination was remarkable for diminished breath sounds at the right lung base, tense abdominal distention, and a positive fluid wave. Echocardiogram revealed a large right-sided pleural effusion and a pericardial effusion. At the time of first presentation, laboratory studies were significant for lymphopenia, elevated sedimentation rate at 95 mm/hour (0–20 mm/hour), elevated C-reactive protein at 35 mg/dL (0–0.8 mg/dL), a ferritin of 120 ng/mL (10–230 ng/mL), a positive anti-nuclear antibody at titer of 1 : 2560, and a positive anti-DNA antibody at a titer of 1 : 640. Her C3 and C4 levels were both decreased at 73 mg/dL (88–201 mg/dL) and <10 mg/dL (16–47 mg/dL), respectively.

**Figure 1:**
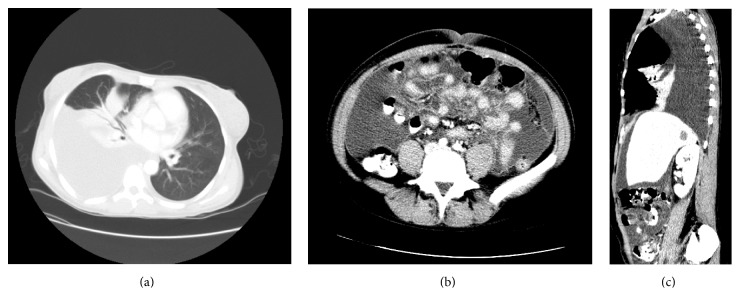
Computerized tomography scan of the abdomen and pelvis in sagittal and transverse planes showing large right-sided pleural effusion and massive abdominal ascites.

The massive ascites prompted a search for an underlying etiology. Routine lab work ruled out cardiac, hepatic, or renal causes. A malignancy workup was then undertaken. Computed tomography of the chest, abdomen, and pelvis was performed, which showed a large right pleural effusion, massive abdominal ascites, and a low-density lesion on the right lobe of the liver (Figures 1(a), 1(b), and 1(c)). CA-125 was elevated at 307 U/mL (<34 U/mL), but carcinoembryonic antigen and alphafetoprotein tumor markers were negative. Subsequent Magnetic Resonance Imaging of the abdomen was done, which revealed the liver lesion to be suspicious for a hemangioma. In the pelvis, right-sided ovarian cysts were visualized which were also not suspicious for malignancy.

**Figure 2:**
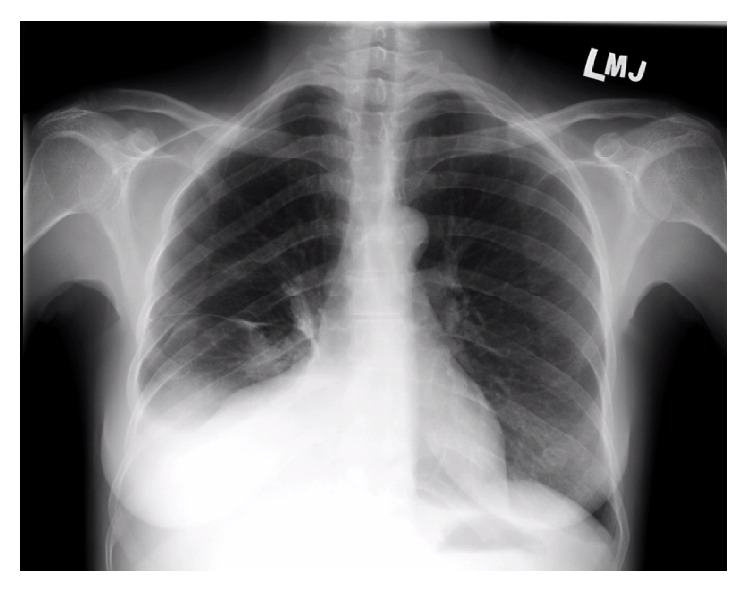
Chest X-ray showing improvement of pleural effusions after abdominal paracentesis, suggesting that the fluid was communicating between pleural and peritoneal cavities.

Multiple diagnostic and therapeutic abdominal paracenteses were done. Fluid analysis on each occasion was suggestive of an exudative ascites with a serum-ascites albumin gradient (SAAG) of 0.3–0.8. No malignant cells were identified. Chest X-rays revealed that pleural effusions improved significantly after paracentesis (Figure 2). The patient was subsequently started on 1 g/day pulse methylprednisolone therapy for 3 days with improvements noted. We suspect the abdominal ascites as a presenting feature in new-onset Systemic Lupus Erythematosus associated Pseudo-Pseudo Meigs Syndrome (PPMS).

## 3. Discussion

The case presented is consistent with the phenomenon known as Pseudo-Pseudo Meigs Syndrome (PPMS). This is defined by the presence of ascites, pleural effusions, and an elevated CA-125 level in a patient with Systemic Lupus Erythematosus (SLE). It was first described by Tjalma in 2005 but since then 6 other articles have been published on the subject (Table 1 [4–10]). As with this instance, the majority of published cases had no prior diagnosis of SLE. We suspect that the stress of her recent surgery triggered an SLE flare and the development of PPMS.

Pseudo-Pseudo Meigs Syndrome must be differentiated from Meigs Syndrome, in which ascites and plural effusions occur in conjunction with a benign ovarian mass (most commonly fibromas, Brenner's tumors, and granulosa cell tumors) and Pseudo-Meigs Syndrome, in which these symptoms develop with tumors other than those originally described. As a criterion for diagnosis of these conditions, symptoms must resolve with excision of the mass. PPMS has no association with either benign or malignant pelvic tumors.

Due to the rarity of the condition, the pathophysiology underlying the ascites of PPMS is still the subject of much debate. It likely represents an SLE phenotype that preferentially involves the serosa. The leading hypotheses suggest severe, uncontrolled inflammation to be the underlying basis of this syndrome. This may be the result of lymphoaggregation of plasma cells, deposition of immune complexes on the peritoneum triggering a local inflammatory reaction or of vasculitis of peritoneal vessels [11]. This inflammatory theory is supported by the finding of high serum ferritin levels in patients with ascites and PPMS [10, 12]. Lee et al. were able to demonstrate ferritin levels >2000 ng/mL (normal 5–204 ng/mL) in 2 patients with PPMS [10] and postulate that hyper expression of this acute phase reactant may point to the underlying basis of this syndrome. In the case presented here, ferritin levels were found to be within normal limits. While this by no means refutes the inflammatory theory, it suggests that the underlying pathophysiology is more complex. Regardless of the principal etiology, ascites in SLE is a peritoneal condition, as supported by SAAG <1.1, and typically results in an exudate. This was reported in every case described in the literature (Table 1).

The elevated CA-125 levels seen in PPMS can also be explained by the inflammation theory. CA-125 has been shown to be constitutionally expressed by the omentum and mesovarium [13] and synthesis is increased when these cells are stimulated as would occur in SLE associated inflammation. This is thought to be the result of upregulation of expression by proinflammatory cytokines such as interleukin- (IL-) 1b, interferon-*γ*, vascular endothelial growth factor (VEGF), and fibroblast growth factor [14], which are themselves induced by the formation and deposition of immune complexes. Publications have also noted a correlation between CA-125 elevation and the amount of ascites, with CA-125 levels dwindling to normal range with the improvement of serositis [15]. CA-125 may therefore be an independent marker for serositis in SLE.

The mechanism of the pleural effusions seen in PPMS is more clear-cut. These likely develop secondary to communication with the peritoneal cavity and mechanical passive transfer of ascetic fluid through diaphragmatic apertures or intracellular gaps or across lymphatic vessels. This is supported by the fact that, in the case described, pleural effusion improved significantly with therapeutic paracentesis.

The general treatment approach to PPMS is to treat the underlying lupus. This usually consists of a pulse dose of steroids followed by a steroid taper. Good results have been reported with this regimen in all the cases analysed [7–10]. In some instances, more potent immunosuppression with azathioprine or cyclophosphamide may be required to control symptoms [4, 10]. Given the inflammatory theory of pathogenesis, it has been postulated that anti-CD20 agents, such as rituximab, which deplete B-cells and decrease immune complex formation, may be more beneficial in relieving ascites in this subset of patients [4, 7]. Recent studies have shown that rituximab is both safe and efficacious for use in refractory SLE [16] but its use in either the broader disease or the PPMS variant has not been established.

## 4. Conclusion

Pseudo-Pseudo Meigs Syndrome is a rare manifestation of SLE. Although the etiology of it is uncertain, its existence is becoming increasingly recognized in the literature. PPMS should therefore be considered in the differential diagnosis of a patient presenting with ascites, pleural effusions, and an elevated CA-125 level and a diagnosis of SLE included in the workup along with the search for malignancy.

## Figures and Tables

**Table 1:** Review of the literature of the reported cases of Pseudo-Pseudo Meigs Syndrome.

Article	Demographic	Prior SLE diagnosis	Presenting symptoms	CA-125 level (<35 U/mL)	Ascites fluid characteristics	Initial treatment
Tjalma 2005 [4]	38F	Yes	Dyspnea, abdominal tenderness	887 U/mL	Exudate	Prednisone, azathioprine

Schmitt et al. 2005 [5]	33F	No	Dyspnea, abdominal distention, pedal edema, poor appetite	1239 U/mL–2287 U/mL	Exudate Cytology negative	Prednisone, mycophenolate mofetil, hydroxychloroquine

Ural et al. 2008 [6]	38F	No	Dyspnea, abdominal distention, rash, skin lesions, alopecia	1229 U/mL	Exudate SAAG < 1.0 AFB negative	Prednisone, hydroxychloroquine

Bes and Soy 2011 [7]	47F	No	Dyspnea, vomiting, diarrhea	233 U/mL	—	Prednisone (40 mg/day)

Dalvi et al. 2012 [8]	56F	Yes	Abdominal distention, poor appetite, weight loss, cachexia	70.1 U/mL	Exudate SAAG < 1.0 Cytology negative	Prednisone (1 mg/kg/day)

Bes et al. 2013 [9]	42F	No	Abdominal pain, abdominal distention, pedal edema, vomiting, diarrhea	91.3 U/mL	Exudate Cytology negative	Methylprednisolone (1 g/d × 3 days)

Lee et al. 2013 [10]	29F	No	Dyspnea, abdominal distention, vomiting	345 U/mL	—	Methylprednisolone (1 g/day × 3 days)
	54F	Yes	Abdominal distention, poor appetite, weight loss, cachexia	344.9 U/mL	SAAG < 1.0 Cytology negative	Methylprednisolone (250 mg/day) Cyclophosphamide (750 mg/day)

McVorran et al. (*current case*) 2016	40F	No	Dyspnea, abdominal distention, Raynaud's phenomenon, arthralgia, photosensitivity	307 U/mL	SAAG < 1.0 Cytology negative	Methylprednisolone (1 g/d × 3 days)

## Additional Points

(i) Pseudo-Pseudo Meigs Syndrome (PPMS) is defined by the presence of ascites, pleural effusions, and an elevated CA-125 level in a patient with Systemic Lupus Erythematosus (SLE). (ii) The leading hypotheses suggest severe, uncontrolled inflammation to be the underlying basis of this syndrome and this is supported by the finding of high serum ferritin levels in these patients. (iii) Anti-CD20 agents may be beneficial in relieving ascites in this subset of patients. (iv) PPMS should be considered in the differential diagnosis of a patient presenting with ascites, pleural effusions, and an elevated CA-125 level.
